# Impact of the COVID‐19 pandemic on TB services at ART programmes in low‐ and middle‐income countries: a multi‐cohort survey

**DOI:** 10.1002/jia2.26018

**Published:** 2022-10-26

**Authors:** Mariana Marti, Kathrin Zürcher, Leslie A. Enane, Lameck Diero, Olivier Marcy, Thierry Tiendrebeogo, Marcel Yotebieng, Christelle Twizere, Suwimon Khusuwan, Evy Yunihastuti, Gary Reubenson, N. Sarita Shah, Matthias Egger, Marie Ballif, Lukas Fenner, the IeDEA global consortium

**Affiliations:** ^1^ Institute of Social and Preventive Medicine University of Bern Bern Switzerland; ^2^ The Ryan White Center for Pediatric Infectious Disease and Global Health Department of Pediatrics Indiana University School of Medicine Indianapolis Indiana USA; ^3^ Department of Medicine Moi University School of Medicine Eldoret Kenya; ^4^ Department of Medicine Moi Teaching and Referral Hospital Eldoret Kenya; ^5^ University of Bordeaux, Inserm U1219 IRD EMR271 Bordeaux France; ^6^ Division of General Internal Medicine Department of Medicine Albert Einstein College of Medicine Bronx New York USA; ^7^ Centre National de Référence en matière de VIH/SIDA Burundi (CNR) Bujumbura Burundi; ^8^ Chiangrai Prachanukroh Hospital Chiang Rai Thailand; ^9^ Faculty of Medicine Universitas Indonesia Dr. Cipto Mangunkusumo General Hospital Jakarta Indonesia; ^10^ Rahima Moosa Mother and Child Hospital Department of Paediatrics and Child Health Faculty of Health Sciences University of the Witwatersrand Johannesburg South Africa; ^11^ Emory Rollins School of Public Health Emory University Atlanta Georgia USA; ^12^ Centre for Infectious Disease Epidemiology and Research Faculty of Health Sciences University of Cape Town Cape Town South Africa; ^13^ Population Health Sciences Bristol Medical School University of Bristol Bristol UK

**Keywords:** tuberculosis, COVID‐19, HIV clinic, antiretroviral therapy, low‐ and middle‐income countries, differentiated service delivery

## Abstract

**Introduction:**

COVID‐19 stretched healthcare systems to their limits, particularly in settings with a pre‐existing high burden of infectious diseases, including HIV and tuberculosis (TB). We studied the impact of COVID‐19 on TB services at antiretroviral therapy (ART) clinics in low‐ and middle‐income countries.

**Methods:**

We surveyed ART clinics providing TB services in the International Epidemiology Databases to Evaluate AIDS (IeDEA) consortium in Africa and the Asia‐Pacific until July 2021 (TB diagnoses until the end of 2021). We collected site‐level data using standardized questionnaires.

**Results:**

Of 46 participating ART clinics, 32 (70%) were in Africa and 14 (30%) in the Asia‐Pacific; 52% provided tertiary care. Most clinics (85%) reported disrupted routine HIV care services during the pandemic, both in Africa (84%) and the Asia‐Pacific (86%). The most frequently reported impacts were on staff (52%) and resource shortages (37%; protective clothing, face masks and disinfectants). Restrictions in TB health services were observed in 12 clinics (26%), mainly reduced access to TB diagnosis and postponed follow‐up visits (6/12, 50% each), and restrictions in TB laboratory services (22%). Restrictions of TB services were addressed by dispensing TB drugs for longer periods than usual (7/12, 58%), providing telehealth services (3/12, 25%) and with changes in directly observed therapy (DOT) (e.g. virtual DOT, 3/12). The number of TB diagnoses at participating clinics decreased by 21% in 2020 compared to 2019; the decline was more pronounced in tertiary than primary/secondary clinics (24% vs. 12%) and in sites from the Asia‐Pacific compared to Africa (46% vs. 14%). In 2021, TB diagnoses continued to decline in Africa (–8%) but not in the Asia‐Pacific (+62%) compared to 2020. During the pandemic, new infection control measures were introduced or intensified at the clinics, including wearing face masks, hand sanitation and patient triage.

**Conclusions:**

The COVID‐19 pandemic led to staff shortages, reduced access to TB care and delays in follow‐up visits for people with TB across IeDEA sites in Africa and the Asia‐Pacific. Increased efforts are needed to restore and secure ongoing access to essential TB services in these contexts.

## 1 INTRODUCTION

Until 2020 when the coronavirus disease 2019 (COVID‐19) pandemic had started, tuberculosis (TB) was the leading infectious cause of death globally. In 2019, an estimated 10 million people developed incident TB, and 1.4 million people died from TB, including 208,000 people with HIV [1]. The emergence of the COVID‐19 pandemic has been a major public health crisis. Globally, more than 5.5 million people were reported to have died from COVID‐19 by the end of 2021 [2], while the estimated mortality reaches 20 million deaths [3]. The COVID‐19 pandemic challenged health systems worldwide, stretching capacities to their limits. The urgent reallocation of resources for COVID‐19 severely disrupted the provision of essential health services, including HIV and TB care, especially in low‐ and middle‐income settings [4, 5].

Although TB incidence and mortality have been steadily declining since 2010, TB care was greatly disrupted during the COVID‐19 pandemic, with reduced access to diagnostics services and delays in treatment initiations [6]. Consequently, the global number of people newly diagnosed with TB dropped by 20% from 2019 to 2020, and TB mortality increased by 5.6%, returning to 2017 levels [6]. Undiagnosed TB can increase the risk of transmission within communities, resulting in secondary cases and potentially increasing TB mortality in the coming years [7, 8]. In addition, TB has been shown to be independently associated with increased mortality among those with COVID‐19 [9], and immunocompromised people, such as people living with HIV, are at higher risk of TB and have worse COVID‐19 outcomes [6, 10, 11].

There is a need to closely monitor the negative effects of the pandemic on TB prevention and care and restore adequate TB care, particularly in high‐TB burden settings and among vulnerable populations, such as people living with HIV [4, 12]. In this study, we analysed trends in the number of newly diagnosed persons affected by TB at antiretroviral therapy (ART) clinics in Africa and the Asia‐Pacific, and the influence of the COVID‐19 pandemic on HIV and TB services in these programmes.

## 2 METHODS

### 2.1 Study setting

**Figure 1:**
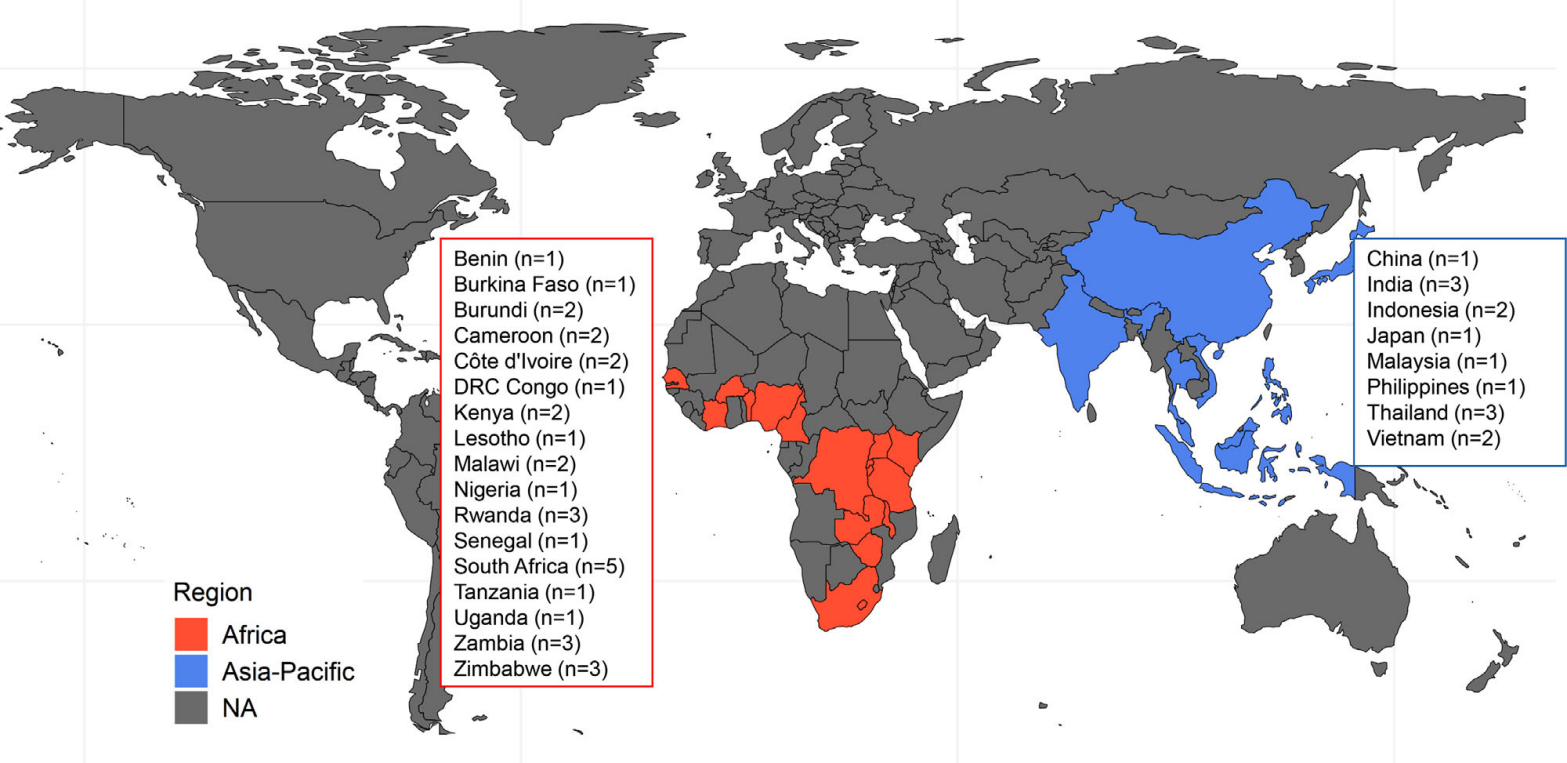
Map of the 46 participating countries and antiretroviral therapy (ART) clinics in the African and the Asian‐Pacific regions of the global International epidemiology Databases to Evaluate AIDS (IeDEA) consortium.

The study was conducted in the International epidemiology Databases to Evaluate AIDS (IeDEA, www.iedea.org) consortium [13]. This consortium was established in 2006 by the U.S. National Institutes of Health to create a rich data resource for HIV‐related research questions. Through centres and research groups in seven geographic regions, observational data are collected on more than 2.2 million people living with or at increased risk for HIV. We included ART clinics offering on‐site TB services (e.g. diagnosis and treatment) to both people living with HIV and HIV‐negative individuals in the five IeDEA regions with high TB incidence in Africa and the Asia‐Pacific (Figure 1).

### 2.2 Data collection

We used a standardized survey tool developed in collaboration with an advisory group of experts in the field of HIV and TB from the local ART clinics and the IeDEA regional data centres. The survey was available in English and French, pilot‐tested in both languages and could be administered via paper forms or electronically. We used REDCap (Research Electronic Data Capture) to collect and manage the survey data [14, 15]. One respondent per clinic completed the survey. Most respondents were physicians (28, 61%), eight (17%) were nurses, six (13%) were clinical officers and four respondents were from other professions. The survey consisted of four components: (A) basic characteristics of the ART clinics (e.g. IeDEA region, country and level of care); (B) information on the impact of COVID‐19 on TB and HIV health and laboratory services; (C) absolute yearly numbers of new TB diagnoses from 2017 to 2021; and (D) information on infection control measures at the ART clinic. Data were collected from July 2021 to January 2022.

### 2.3 Definitions

A TB diagnosis was defined as either being bacteriologically confirmed (positive by smear microscopy, culture, lateral‐flow urine tests or rapid diagnostic tests, such as Xpert MTB/RIF or line probe assay) or clinically diagnosed at the ART clinic and the associated TB services [16]. We assessed disruptions at ART clinics as well as infection control measures from the beginning of the COVID‐19 pandemic until July 2021. We classified infection control measures within the following categories, based on the World Health Organization (WHO) guidelines [17]: “screening and triage,” “precautions for patients” (e.g. hand and respiratory hygiene), “administrative controls” (e.g. administrative measures to manage visitors) and “engineering and environmental controls” (e.g. physical distancing and ventilation). We added a category “precautions for staff members.” Disruptions in any TB‐related services were defined as either shortage of anti‐TB drugs, TB laboratory services or restrictions in TB health services. A delay in TB treatment initiation was defined as a delay caused by a pandemic‐related cause (e.g. travel restrictions). Directly observed therapy (DOT) was defined as when a community volunteer or trained family member routinely observes participants taking their medication to improve adherence [18].

### 2.4 Statistical analyses

We used descriptive statistics to assess the changes in availability and utilization of health services, resources and infection control measures at the ART clinics during the COVID‐19 pandemic. We described the yearly number of TB diagnoses with the previous year, overall, by region and by care level (primary/secondary and tertiary). The analyses were performed in Stata version 16.0 (Stata Corporation, College Station, TX, USA) or in R (version 3.6.0).

### 2.5 Ethics statement

All participating IeDEA sites obtained approvals from their local institutional review boards or ethics committees to participate in IeDEA research. All participants provided informed consent before participating. In addition, the Cantonal Ethics Committee of Bern (Switzerland) approved the project.

## 3 RESULTS

### 3.1 Participating sites

We collected data from 46 ART clinics across 25 countries. Thirty‐two (70%) clinics were in Africa and 14 (30%) in the Asia‐Pacific region of IeDEA (Figure 1 and Table S1). Most of the clinics were in urban settings (37, 80%), with seven (15%) in peri‐urban and two (4%) in rural settings. Twenty‐four (52%) were tertiary care clinics, five (11%) secondary and 17 (37%) primary care clinics. Twenty‐three (51%) clinics treated both inpatients and outpatients, 20 (44%) outpatients only and two (4%) inpatients only. The majority of ART clinics (41, 89%) reported screening for symptoms of COVID‐19 during routine consultations (Table S2). COVID‐19 testing was performed in 36 (78%) clinics, most commonly with nasopharyngeal swab polymerase chain reaction (29, 63%) and rapid antigen testing (24, 52%). National COVID‐19 vaccination campaigns had begun in 44 clinics (96%) at the time of the survey.

### 3.2 Impact of COVID‐19 on participating ART clinics

**Figure 2:**
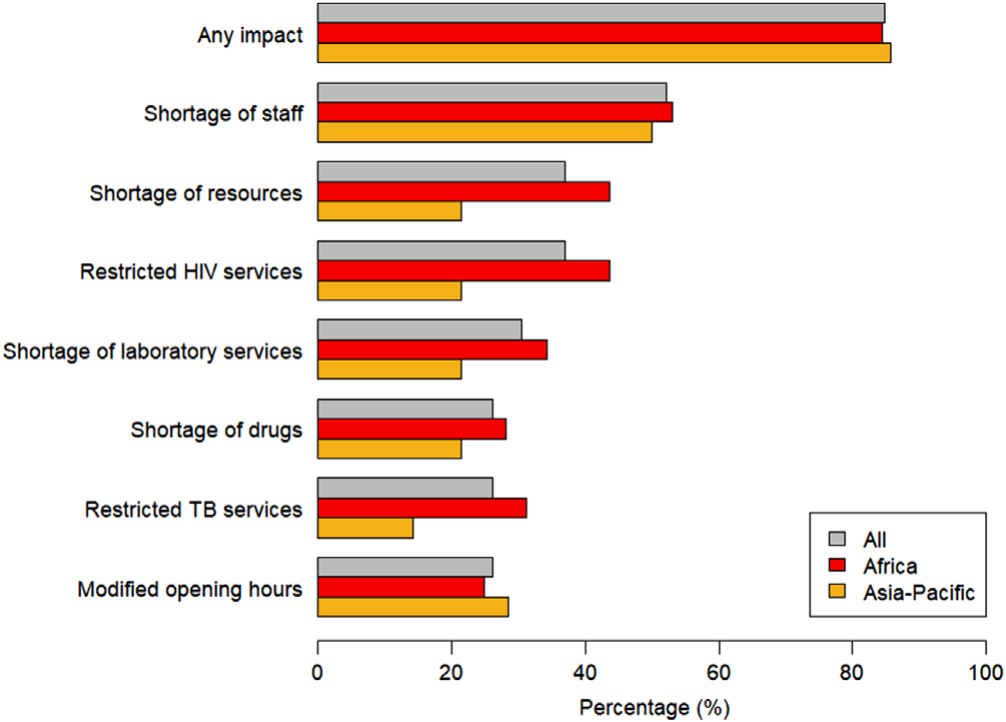
Impact of the COVID‐19 pandemic on health services at 46 ART clinics, overall and by region. Abbreviation: TB, tuberculosis.

Most ART clinics (39/46, 85%) reported that their routine HIV care services were affected, both in Africa (27/32, 84%) and the Asia‐Pacific (12/14, 86%; Figure 2). More tertiary clinics were affected than primary/secondary clinics (22/24, 92% vs. 17/22, 77%). The most frequently observed impact was staff shortages (24/46, 52%), which was greatest among nurses (22/24, 92%), followed by medical doctors and laboratory technicians (both 10/24, 42%). Staff shortages were primarily caused by staff isolation/quarantine (17/24, 71%) and reassignments to COVID‐related services (13/24, 54%). The next most frequent impacts were shortages of resources, most commonly protective clothing, face masks, disinfectants and restricted HIV services (both 17/46, 37%). Some clinics had to modify their opening hours (10/46, 22%), but none of them closed entirely during the pandemic (Table 1). The distribution of reported impacts varied across clinics (Figure S1).

**Table 1:** Overview of the reported shortages or restrictions at the ART clinics, by region and service levels

		Region		Care level	
	Total	Africa	Asia‐Pacific	Primary/secondary	Tertiary
Category, *n* (%)	(*n* = 46)	(*n* = 32)	(*n* = 14)	(*n* = 22)	(*n* = 24)
Shortage of staff					
No	22 (48)	15 (47)	7 (50)	11 (50)	11 (46)
Yes	24 (52)	17 (53)	7 (50)	11 (50)	13 (54)
Nurses	22 (48)	16 (50)	6 (43)	10 (45)	11 (46)
Medical doctors	10 (22)	7 (22)	3 (21)	4 (18)	6 (25)
Laboratory technicians	10 (22)	8 (25)	2 (14)	5 (23)	4 (17)
Clinical officers	6 (13)	6 (19)	0 (0)	3 (14)	3 (13)
Pharmacists	7 (15)	6 (19)	1 (7)	4 (18)	2 (8)
Restricted HIV services					
No	19 (63)	18 (56)	11 (79)	15 (68)	14 (58)
Yes	17 (37)	14 (44)	3 (21)	7 (32)	10 (42)
Postponed follow‐up visits	9 (20)	8 (25)	1 (7)	3 (14)	6 (25)
Reduced access to HIV diagnosis	5 (11)	4 (13)	1 (7)	2 (9)	3 (13)
Delay in HIV treatment start	4 (9)	4 (13)	0 (0)	1 (5)	3 (13)
Shortage of resources					
No	29 (63)	18 (56)	11 (79)	10 (45)	19 (79)
Yes	17 (37)	14 (44)	3 (21)	12 (55)	5 (21)
Protective clothes	12 (26)	11 (34)	1 (7)	9 (41)	3 (13)
Face masks	11 (24)	10 (31)	1 (7)	8 (36)	3 (13)
Disinfectant	8 (17)	7 (22)	1 (7)	5 (23)	3 (13)
Face shields	7 (15)	6 (19)	1 (7)	5 (23)	2 (8)
Protective gloves	5 (11)	4 (13)	1 (7)	4 (18)	1 (4)
Safety glasses	4 (9)	4 (13)	0 (0)	4 (18)	0 (0)
Surgical hoods	4 (9)	4 (13)	0 (0)	4 (18)	0 (0)
Intravenous material	3 (7)	3 (9)	0 (0)	3 (14)	0 (0)
Equipment for oxygen units	3 (7)	3 (9)	0 (0)	2 (9)	1 (4)
Oxygen bottle	3 (7)	3 (9)	0 (0)	2 (9)	1 (4)
No	32 (70)	21 (66)	11 (79)	15 (68)	17 (71)
Yes	14 (30)	11 (34)	3 (21)	7 (32)	7 (29)
HIV viral load	11 (24)	10 (31)	1 (7)	5 (23)	6 (25)
HIV CD4 cell count	7 (15)	5 (16)	2 (14)	3 (14)	4 (17)
HIV diagnosis	4 (9)	3 (9)	1 (7)	1 (5)	3 (13)
No	34 (74)	24 (75)	10 (71)	16 (73)	18 (75)
Yes	12 (26)	8 (25)	4 (29)	6 (27)	6 (25)
The clinic operated on abbreviated hours	10 (22)	6 (19)	4 (29)	5 (23)	5 (21)
The clinic was open only for emergencies	2 (4)	1 (3)	1 (7)	1 (5)	1 (4)
Shortage of drugs					
No	34 (74)	23 (72)	11 (79)	13 (59)	21 (87)
Yes	12 (26)	9 (28)	3 (21)	9 (41)	3 (13)
Frist‐line ARV	6 (13)	4 (13)	2 (14)	4 (18)	2 (8)
Second‐line ARV	6 (13)	4 (13)	2 (14)	4 (18)	2 (8)
Other antibiotics (incl. Cotrimoxazol)	5 (11)	5 (16)	0 (0)	4 (18)	1 (4)
PrEP	3 (7)	3 (9)	0 (0)	3 (14)	0 (0)

Note: Tuberculosis‐related services are shown in Table 2.

Abbreviation: ARV, antiretroviral drug; PrEP, pre‐exposure prophylaxis.

### 3.3 Impact of COVID‐19 on TB services at the ART clinics

Experiencing any disruption in TB‐related services (health services and laboratory services) was reported by 17 (37%) clinics (Table 2). Laboratory services for TB diagnosis were restricted at 10 (22%) clinics, with reduced access to Xpert MTB/RIF (8, 17%), TB cultures (5, 11%), and line probe assays (5, 11%) being the most frequent restricted services. There were delays in TB treatment initiation reported by four (9%) clinics; follow‐up visits had to be postponed at six (13%) clinics and two (4%) clinics reported shortages in first‐ or second‐line anti‐TB medicines.

**Table 2:** Impact on tuberculosis (TB) health services and TB‐related resources (laboratory services and anti‐TB drugs) at the ART clinics and how disruptions in TB health services were addressed

		Region		Care level	
	Total	Africa	Asia‐Pacific	Primary/secondary	Tertiary
Category, *n* (%)	(*n* = 46)	(*n* = 32)	(*n* = 14)	(*n* = 22)	(*n* = 24)
Any disruptions in TB‐related services^a^					
No	29 (64)	19 (59)	10 (71)	12 (52)	17 (59)
Yes	17 (36)	13 (41)	4 (29)	10 (48)	7 (41)
Restricted TB health services					
No	34 (74)	22 (69)	12 (86)	16 (73)	18 (75)
Yes	12 (26)	10 (31)	2 (14)	6 (27)	6 (25)
Reduced access to TB care/diagnosis	6 (13)	5 (16)	1 (7)	4 (18)	2 (8)
Postponed follow‐up visits	6 (13)	5 (16)	1 (7)	2 (9)	4 (17)
Delay in TB treatment start	4 (9)	4 (13)	0 (0)	3 (14)	1 (4)
Restrictions in contact tracing	4 (9)	3 (9)	1 (7)	2 (9)	2 (8)
How were disruptions in TB health services addressed?					
TB drugs dispensed for longer periods	7/12 (58)	5/10 (16)	2/2 (100)	3/6 (50)	4/6 (67)
Providing telehealth services	3/12 (25)	2/10 (20)	1/2 (50)	1/6 (17)	2/6 (33)
Changes in the DOT provision	3/12 (25)	3/10 (33)	0 (0)	2/6 (33)	1/6 (17)
Restricted TB laboratory services					
No	36 (78)	25 (78)	11 (79)	16 (73)	20 (83)
Yes	10 (22)	7 (22)	3 (21)	6 (27)	4 (17)
GeneXpert for TB	8 (17)	5 (16)	3 (21)	5 (23)	3 (13)
TB culture	5 (11)	3 (9)	2 (14)	3 (14)	2 (8)
Line probe assay for TB^b^	5 (11)	4 (13)	1 (7)	3 (14)	2 (8)
Tuberculin skin testing	4 (9)	3 (9)	1 (7)	3 (14)	1 (4)
NAATs for TB	3 (7)	2 (6)	1 (7)	2 (9)	1 (4)
TB smear microscopy	2 (4)	2 (6)	0 (0)	1 (5)	1 (4)
Urine LAM for TB	2 (4)	1 (3)	1 (7)	2 (9)	0 (0)
Shortages of anti‐TB drugs					
No	44 (96)	30 (94)	0 (0)	20 (91)	24 (100)
Yes	2 (4)	2 (6)	0 (0)	2 (9)	0 (0)
Second‐line anti‐TB drugs	2 (4)	2 (6)	0 (0)	2 (9)	0 (0)
First‐line anti‐TB drugs	2 (4)	2 (6)	0 (0)	2 (9)	0 (0)

Abbreviations: DOT, directly observed therapy; NAATs, nucleic acid amplification tests; TB, tuberculosis; Urin LAM, urine lipoarabinomannan assay.

^a^ Defined as any restriction in TB health services, TB laboratory services or shortage of anti‐TB drugs.

^b^ Hain Genotype MTBDRplus or Genotype MTBDRsl.

Among the 12 clinics that reported restrictions in TB services, seven (58%) provided extended prescriptions of anti‐TB treatment to last for longer periods, three (25%) clinics strengthened telehealth TB services in place of regular consultations, to manage treatment side effects, or to prescribe medications, and three (25%) clinics reduced face‐to‐face DOT with clinic staff (Table 2).

### 3.4 Impact of COVID‐19 on the number of TB diagnoses at ART clinics

**Figure 3:**
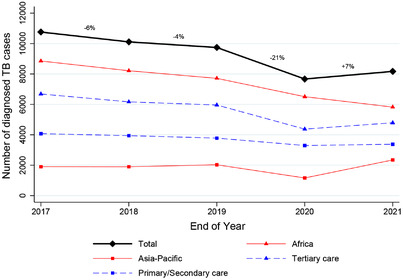
Absolute number of tuberculosis (TB) diagnoses at ART clinics per year between 2017 and 2021. Percentages of the black line (total) indicate the changes from one year to the next in percentage. See also Table S3 with absolute numbers and percentages for each category.

The yearly numbers of new TB diagnoses were available for 37 of the 46 participating clinics (missing for one clinic in the Asia‐Pacific region and eight clinics in Africa). Overall, TB diagnoses decreased by –9% in 2019 compared to 2017 (from 10,758 to 9743). In 2020, TB diagnoses dropped by –21% compared to 2019 (Figure 3). After that, the number increased again, from 7670 in 2020 to 8170 in 2021 (+5%) but did not reach the pre‐pandemic level (76% of the 2017 figure). The decline in newly diagnosed TB from 2019 to 2020 was more pronounced in tertiary care clinics than primary or secondary clinics (–24% vs. –12%) and at sites within the Asia‐Pacific region compared to the African regions of IeDEA (–46% vs. –14%; Table S3). From 2020 to 2021, TB diagnoses continued to decline in Africa from 6508 to 5823 persons affected by TB (–8%). In contrast, TB diagnoses increased at sites in the Asia‐Pacific, from 1162 in 2020 to 2347 in 2021 (+62%), exceeding those of 2017.

### 3.5 Introduction of infection control measures due to the COVID‐19 pandemic at ART clinics

**Figure 4:**
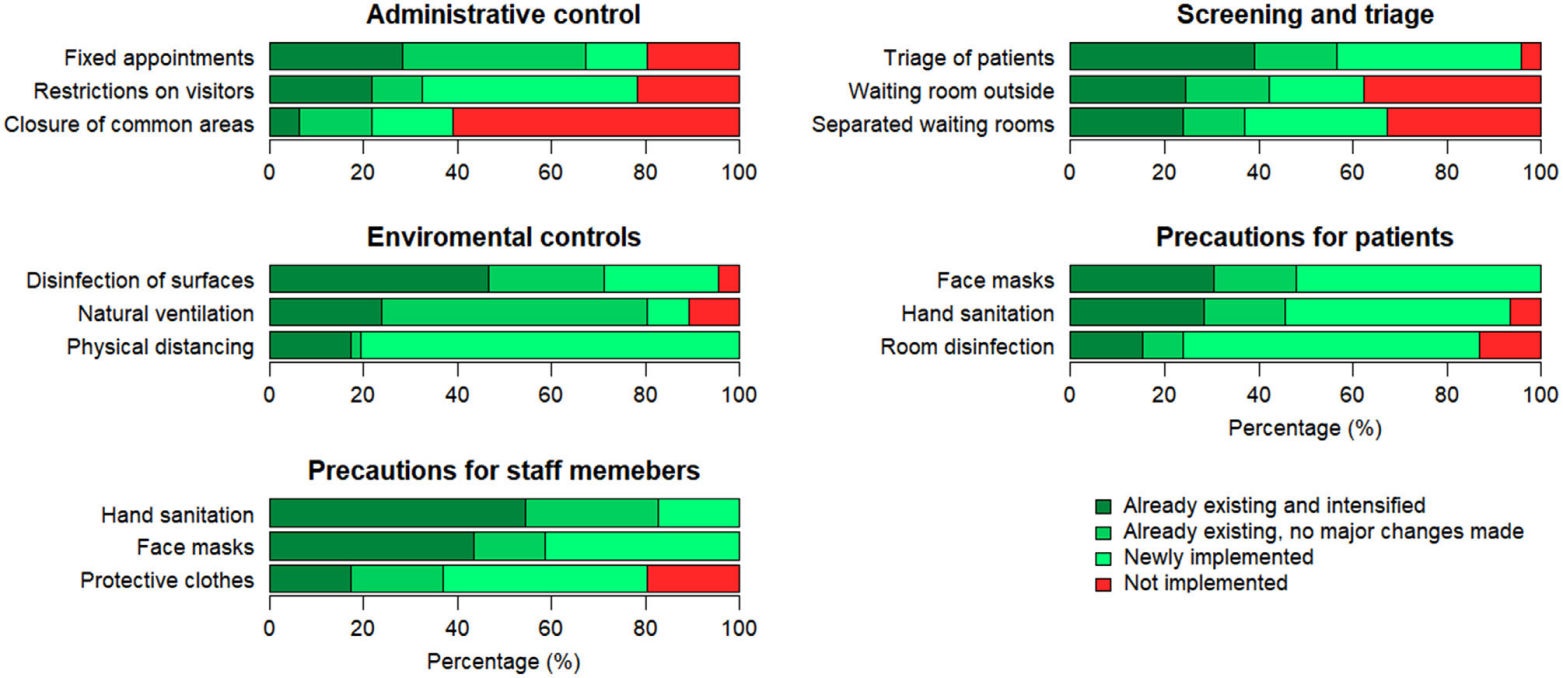
Overview of infection control measures that were newly implemented, intensified, already resisting or not implemented at the ART clinics during the COVID‐19 pandemic (as of July 2021), by World Health Organization (WHO) categories for infection control measures.

During the pandemic, the participating ART clinics reported strengthening their infection control strategies (Figure 4 and Table S4). Twenty‐one (46%) clinics introduced restrictions on visitors to limit the number of persons present. In addition, fixed appointments to reduce waiting times were newly implemented in six clinics (13%). Fourteen (30%) clinics introduced separate waiting rooms for patients with respiratory symptoms, and nine clinics (20%) placed their waiting rooms outside. Patient triage approaches were newly implemented or intensified at 36 (78%) clinics. Mandatory face masks for patients aged ≥ 12 years were introduced in 26 (57%) clinics, and for staff in 27 (59%) clinics. The remaining clinics recommended face masks without making them mandatory (Figure 4 and Figure S2). Face shields for staff were implemented at 25 (54%) clinics. Clinics also implemented environmental protective measures, including physical distancing at 37 (80%) clinics and intensified natural ventilation at 11 clinics (24%).

## 4 DISCUSSION

COVID‐19 has resulted in millions of deaths worldwide and has stretched health systems to their limits, often with important restrictions on routine services. Fragile health systems and under‐resourced countries with a pre‐existing high burden of infectious diseases, including HIV and TB, are particularly vulnerable in this context. In this study, we surveyed 46 ART clinics providing TB services in Africa and Asia‐Pacific to assess how COVID‐19 affected HIV services in general, as well as TB diagnosis and care. We found that almost 90% of the participating ART clinics reported some disruptions in their routine health services during the COVID‐19 pandemic. About 40% reported reductions in their essential TB services, including reduced access to TB diagnostics. The reduced access to care and diagnosis likely led to the observed decrease in the number of new TB diagnoses in 2020 compared to 2019. The decline in TB diagnoses was particularly marked in tertiary care clinics, possibly reflecting the important role those larger hospitals had in diagnosing and treating COVID‐19 cases.

Emergent epidemics and pandemics have been shown to affect healthcare systems and health services with both acute and long‐lasting effects [19]. For example, during the 2014 Ebola epidemic in West Africa, care for people living with HIV/AIDS, malaria or TB was disrupted and mortality rates rose [20, 21]. In 2021, WHO reported a substantial global decline in notified TB diagnoses attributed to the COVID‐19 pandemic, setting back years of progress in TB prevention and care [4, 6]. A modelling study in India, Kenya and Ukraine showed that even short lockdowns during the COVID‐19 pandemic could cause an increase of up to 9% in TB incidence and up to 16% in TB mortality in the proceeding 5 years due to the undetected TB and continued transmission [8]. This threat underscores the urgent need to restore TB care and avoid long‐lasting deteriorations in TB prevention and care.

Staff shortages have been a significant hallmark of the COVID‐19 pandemic [10]. We found that half of participating clinics experienced shortages of staff during the pandemic, either due to isolation or quarantine or because services were reorganized to take care of the rapidly increasing hospitalization of COVID‐19 patients. Nurses, who are often on the frontline of care, were most often absent, demonstrating the negative consequences of not prioritizing healthcare workers for vaccines and allocating resources for personal protective equipment. Infection control measures play a key role in reducing SARS‐CoV‐2 transmission in health facilities, including administrative, environmental and personal protection measures [17]. Infection control measures targeted at reducing transmission may also reduce transmission of airborne diseases, including TB. A modelling study showed that transmission of TB between clinic attendees can be reduced by 83% using outdoor waiting areas, 55% by opening windows and doors, and 47% by mandatory wearing of facemasks for attendees and healthcare workers [22].

We observed that clinics reporting disruptions in TB health services addressed the restrictions by dispensing TB drugs for longer periods than usual in more than half of the sites and providing telehealth services and changes in DOT (e.g. use of virtual DOT) in a few sites. The COVID‐19 pandemic highlighted opportunities to strengthen differentiated services in TB care and to expand the use of digital technologies, as a way to better address individual needs and reduce TB‐associated stigma [11, 23, 24]. These advantages have been documented for HIV care delivery and should be expanded to TB services [10]. In addition, COVID‐19 provided opportunities to introduce stringent infection control measures in the clinics to reduce the risk of airborne diseases, including TB. The pandemic also encouraged patient‐centred differentiated service delivery options to maintain access to care while minimizing physical contact at the clinics [25]. Approaches included the provision of longer drug prescriptions and the availability of telehealth services.

Our cross‐sectional study design limited our ability to capture changes in the measures undertaken to control the spread of SARS‐CoV‐2 over the epidemic waves. It did allow us to rapidly and simultaneously collect data collection within a global consortium of ART clinics using a standardized questionnaire, thus providing timely and comparable data on the impact of COVID‐19 in these regions. We also did not collect detailed patient‐level data, which would have provided valuable information on the impact of COVID‐19 on clinical outcomes, such as TB treatment outcomes and mortality, or differences by gender or age group [26]. Our data showed that fewer new TB diagnoses were documented. We suspect that one of the main reasons for that was a reduction in TB testing, but unfortunately, data on the number of TB diagnostics performed (e.g. number of tests performed) were unavailable. In addition, the observed reduction in TB diagnoses may also reflect data collection issues or changes in health‐seeking behaviours. Finally, we did not assess clinicians’ and patients’ access to vaccines against COVID‐19 since national campaigns were only starting to be available at the time of data collection.

## 5 CONCLUSIONS

Our study showed that the COVID‐19 pandemic disrupted essential services at ART clinics in Africa and the Asia‐Pacific. A marked decline in TB diagnoses was observed from 2019 to 2020, likely related to limited access to care and diagnostics; numbers were increasing again in 2021 in Asia‐Pacific but not in Africa. There is an urgent need to reverse these trends by ensuring sustained investments in TB prevention and care. The COVID‐19 pandemic provides momentum to accelerate digital health and reassess TB service delivery approaches [3], including the provision of telehealth services or longer drug dispensing. Increased efforts are now needed to mitigate the disruptions of essential TB services, restore drug supply chains, support the healthcare workforce and adapt TB service delivery models. These measures should be undertaken in a joint effort, combining resources to tackle TB, COVID‐19, HIV, as well as other infectious diseases [26]. In the future, it is critical that more convenient and patient‐centred approaches, as well as proven infection control measures, will be continued and strengthened, even beyond the pandemic. Moreover, increased focus on screening for respiratory symptoms of COVID‐19 could have been an opportunity for increasing TB screening but was often a missed opportunity. Future disruptions of and adaptations to TB diagnosis and care delivery, as well as their impact on the TB care cascade, need to be closely monitored.

## COMPETING INTERESTS

None to declare.

## AUTHORS’ CONTRIBUTIONS

Conception and design: KZ, MB and LF. MM, KZ, LAE, ME, MB and LF drafted the questionnaires. MM and KZ incorporated the questionnaire into REDCap. MM and KZ analysed the data. MM, KZ and MB wrote the first draft. All authors provided input into the study design, on the questionnaires, analyses and drafting of the paper. All authors reviewed and approved the final version of the manuscript.

## FUNDING

IeDEA is supported by the U.S. National Institutes of Health's National Institute of Allergy and Infectious Diseases, the *Eunice Kennedy Shriver* National Institute of Child Health and Human Development, the National Cancer Institute, the National Institute of Mental Health, the National Institute on Drug Abuse, the National Heart, Lung, and Blood Institute, the National Institute on Alcohol Abuse and Alcoholism, the National Institute of Diabetes and Digestive and Kidney Diseases, the Fogarty International Center and the National Library of Medicine: Asia‐Pacific, U01AI069907; CCASAnet, U01AI069923; Central Africa, U01AI096299; East Africa, U01AI069911; NA‐ACCORD, U01AI069918; Southern Africa, U01AI069924; West Africa, U01AI069919. Informatics resources are supported by the Harmonist project, R24AI24872. LE is supported by the *Eunice Kennedy Shriver* National Institute of Child Health and Human Development under award number K23HD095778. ME was supported by special project funding (grant 189498) from the Swiss National Science Foundation.

## DISCLAIMER

This work is solely the responsibility of the authors and does not necessarily represent the official views of any of the institutions mentioned.

## Supporting information


**Figure S1**: Detailed overview of impacts on any health services and resources during the COVID‐19 pandemic at the 46 participating ART clinics (situation as of July 2021).
**Figure S2**: Detailed overview of control measures for staff and patients.
**Table S1**: Site description of the 46 participating antiretroviral therapy (ART) clinics offering tuberculosis services.
**Table S2**: Diagnostic practices of SARS‐CoV‐2 at the ART clinics (situation as of July 2021).
**Table S3**: Trends in tuberculosis (TB) diagnoses over time (based on data from 37/46 participating clinics).
**Table S4**: Overview of infection control measures at the ART clinics.

## Data Availability

Complete data for this study cannot be posted in a supplemental file or a public repository because of legal and ethical restrictions. The Principles of Collaboration of this multi‐national consortium and the regulatory requirements of the different countries’ IRBs require the submission and approval of individual project concept sheets that describe the planned analyses. Specifically, while the data held by the IeDEA consortium may be available to other investigators, the proposed use must be based on a concept note that is approved by the regional Steering Groups and the IeDEA Executive Committee (Chairperson: Annette Sohn, MD; email: annette.sohn@treatasia.org).
