# Supplementary material for: Impact of the COVID‐19 pandemic on TB services at ART programmes in low‐ and middle‐income countries: a multi‐cohort survey
Source: J Int AIDS Soc. 2022 Oct 26;25(10):e26018. doi: 10.1002/jia2.26018 (PMC9597377; doi:10.1002/jia2.26018)

**SUPPLEMENTARY INFORMATION**

**Supplementary Table 1:** Site description of the 46 participating Antiretroviral Therapy (ART) clinics offering tuberculosis services.

| **Characteristics, n (%)** | **Total**  **(n=46)** | **Africa**  **(n=32)** | **Asia-Pacific**  **(n=14)** |
| --- | --- | --- | --- |
| **Setting** |  |  |  |
| Urban | 37 (80) | 23 (72) | 14 (100) |
| Peri-urban (immediately adjoining urban areas) | 7 (15) | 7 (22) | 0 (0) |
| Rural | 2 (4) | 2 (4) | 0 (0) |
| **Care level** |  |  |  |
| Primary (health care centre or facility) | 17 (37) | 15 (47) | 2 (14) |
| Secondary (district or provincial hospital) | 5 (11) | 5 (16) | 0 (0) |
| Tertiary (teaching or referral hospital) | 24 (52) | 12 (38) | 12 (86) |
| **Adults and/or children care** |  |  |  |
| Adults only | 10 (22) | 4 (13) | 6 (43) |
| Both adults and children | 36 (78) | 28 (88) | 8 (57) |
| **In and/or out-patients treatment** |  |  |  |
| In-patients only | 2 (4) | 1 (3) | 1 (7) |
| out-patients only | 21 (45) | 16 (49) | 5 (36) |
| Both in- and out-patients | 23 (51) | 15 (48) | 8 (57) |

**Supplementary Table 2:** Diagnostic practices of SARS-CoV-2 at the ART clinics (situation as of July 2021).

|  |  |  | **Region** | |  | **Care level** | | |
| --- | --- | --- | --- | --- | --- | --- | --- | --- |
| **Category, n (%)** | **Total**  **(n=46)** |  | **Africa**  **(n=32)** | **Asia-Pacific**  **(n=14)** |  | **Primary and secondary**  **(n=22)** | **Tertiary**  **(n=24)** | |
| **Screening** |  |  |  |  |  |  |  |  |
| No | 5 (11) |  | 4 (13) | 1 (7) |  | 2 (14) | 2 (8) |  |
| Yes | 41 (89) |  | 28 (88) | 13 (93) |  | 19 (86) | 22 (92) |  |
| *Everyone* | *29 (63)* |  | *19 (59)* | *10 (71)* |  | *15 (68)* | *14 (58)* |  |
| *People with COVID-19 symptoms* | *12 (26)* |  | *9 (28)* | *3 (21)* |  | *4 (18)* | *8 (33)* |  |
| *Close contacts* | *5 (12)* |  | *5 (16)* | *0 (0)* |  | *3 (14)* | *2 (8)* |  |
| **SARS-CoV-2 diagnostic test used** | | |  |  |  |  |  |  |
| No | 10 (22) |  | 7 (22) | 3 (21) |  | 5 (23) | 5 (21) |  |
| Yes | 36 (78) |  | 25 (78) | 11 (79) |  | 17 (77) | 19 (79) |  |
| *PCR (nasopharyngeal swab)* | *29 (63)* |  | *18 (56)* | *11 (79)* |  | *10 (45)* | *19 (79)* |  |
| *Rapid antigen tests (antigen-RDT)* | *24 (52)* |  | *15 (47)* | *9 (64)* |  | *11 (50)* | *13 (54)* |  |
| *Symptom based* | *14 (30)* |  | *11 (34)* | *3 (21)* |  | *7 (32)* | *7 (29)* |  |
| *Imaging (X-ray, CT scan, ultrasound)* | *11 (24)* |  | *6 (19)* | *5 (36)* |  | *4 (18)* | *7 (29)* |  |
| *Other nucleic acid amplification tests* | *6 (13)* |  | *1 (3)* | *5 (36)* |  | *0 (0)* | *6 (25)* |  |
| *Serology / Antibody detection** | *6 (13)* |  | *4 (13)* | *2 (14)* |  | *3 (14)* | *3 (13)* |  |
| **Communication of test results** | | | |  |  |  |  |  |
| *By phone call* | *18 (39)* |  | *11 (34)* | *7 (50)* |  | *6 (27)* | *12 (50)* |  |
| *By text message* | *12 (26)* |  | *8 (25)* | *4 (29)* |  | *5 (23)* | *7 (29)* |  |
| *By e-mail* | *3 (7)* |  | *1 (3)* | *2 (14)* |  | *0 (0)* | *3 (13)* |  |
| *During a visit at the clinic* | *22 (48)* |  | *16 (50)* | *6 (43)* |  | *12 (55)* | *10 (42)* |  |
| *Patients accessed electronically* | *2 (4)* |  | *3 (9)* | *0 (0)* |  | *1 (5)* | *2 (8)* |  |
| **All presumptive persons affected by TB screened for COVID-19** | |  |  |  |  |  |  |  |
| No | 31 (67) |  | 20 (63) | 11 (79) |  | 17 (77) | 12 (50) |  |
| Yes | 15 (33) |  | 12 (38) | 3 (21) |  | 5 (23) | 12 (50) |  |

Abbreviations: PCR, polymerase chain reaction; RDT, rapid diagnostic test; CT, computer tomography

* Antibody tests are not suitable for detecting acute infection because they cannot distinguish between ongoing or old infections or vaccinations.

**Supplementary Table 3:** Trends in tuberculosis (TB) diagnoses over time (based on data from 37/46 participating clinics). n indicates absolute numbers and % indicate percent changes from one year to the next (+, positive; -, negative).

|  | 2017 |  | 2018 | |  | 2019 | |  | 2020 | |  | 2021 | |
| --- | --- | --- | --- | --- | --- | --- | --- | --- | --- | --- | --- | --- | --- |
|  | n |  | n | % |  | n | % |  | n | % |  | n | % |
| **Total** | 10,758 |  | 10,112 | -6% |  | 9,743 | -4% |  | 7,670 | -21% |  | 8,170 | +7% |
| **Region** |  |  |  |  |  |  |  |  |  |  |  |  |  |
| Africa | 8,853 |  | 8,214 | -7% |  | 7,714 | -6% |  | 6,508 | -16% |  | 5,823 | -11% |
| Asia-Pacific | 1,905 |  | 1,898 | 0% |  | 2,029 | +7% |  | 1,162 | -43% |  | 2,347 | +102% |
| **Care level** |  |  |  |  |  |  |  |  |  |  |  |  |  |
| Primary/secondary | 4,074 |  | 3,945 | -3% |  | 3,783 | -4% |  | 3,296 | -13% |  | 3,379 | +3% |
| Tertiary | 6,684 |  | 6,167 | -8% |  | 5,960 | -3% |  | 4,374 | -27% |  | 4,791 | +10% |

**Supplementary Table 4:** Overview of infection control measures at the ART clinics.

| **Category, n (%)** | | Newly implemented | Already existing and intensified | Already existing, no major changes made | Not implemented |
| --- | --- | --- | --- | --- | --- |
| **Screening and triage** | |  |  |  |  |
| **Triage of patients** | |  |  |  |  |
| Overall (n=46) | | 18 (39) | 18 (39) | 8 (17) | 2 (4) |
| Region | Africa (n=32) | 12 (38) | 12 (38) | 7 (22) | 1 (3) |
|  | Asia-Pacific (n=14) | 6 (43) | 6 (43) | 1 (7) | 1 (7) |
| Service level | Primary/secondary (n=22) | 9 (41) | 9 (41) | 4 (18) | 0 (0) |
|  | Tertiary (n=24) | 9 (38) | 9 (38) | 4 (17) | 2 (8) |
| **Separated waiting rooms** | | |  |  |  |
| Overall (n=46) | | 14 (30) | 11 (24) | 6 (13) | 15 (33) |
| Region | Africa (n=32) | 9 (28) | 6 (19) | 5 (16) | 12 (38) |
|  | Asia-Pacific (n=14) | 5 (36) | 5 (36) | 1 (7) | 3 (21) |
| Service level | Primary/secondary (n=22) | 8 (36) | 4 (18) | 2 (9) | 8 (36) |
|  | Tertiary (n=24) | 6 (25) | 7 (29) | 4 (17) | 7 (29) |
| **Waiting room outside** | | |  |  |  |
| Overall (n=46) | | 9 (20) | 11 (24) | 8 (18) | 17 (38) |
| Region | Africa (n=32) | 5 (16) | 7 (22) | 6 (19) | 14 (44) |
|  | Asia-Pacific (n=14) | 4 (31) | 4 (31) | 2 (15) | 3 (23) |
| Service level | Primary/secondary (n=22) | 6 (29) | 5 (24) | 3 (14) | 7 (33) |
|  | Tertiary (n=24) | 3 (13) | 6 (25) | 5 (21) | 10 (42) |
| **Administrative controls** | | |  |  |  |
| **Restrictions on visitors** | | |  |  |  |
| Overall (n=46) | | 21 (46) | 10 (22) | 5 (11) | 10 (22) |
| Region | Africa (n=32) | 15 (47) | 4 (13) | 3 (9) | 10 (31) |
|  | Asia-Pacific (n=14) | 6 (43) | 6 (43) | 2 (14) | 0 (0) |
| Service level | Primary/secondary (n=22) | 10 (45) | 3 (14) | 3 (14) | 6 (27) |
|  | Tertiary (n=24) | 11 (46) | 7 (29) | 2 (8) | 4 (17) |
| **Closure of common areas in clinic premises** | | |  |  |  |
| Overall (n=46) | | 8 (17) | 3 (7) | 7 (15) | 28 (61) |
| Region | Africa (n=32) | 4 (13) | 0 (0) | 5 (16) | 23 (72) |
|  | Asia-Pacific (n=14) | 4 (29) | 3 (21) | 2 (14) | 5 (36) |
| Service level | Primary/secondary (n=22) | 2 (9) | 1 (5) | 3 (14) | 16 (73) |
|  | Tertiary (n=24) | 6 (25) | 2 (8) | 4 (17) | 15 (50) |
| **Fixed appointments** | | |  |  |  |
| Overall (n=46) | | 6 (13) | 13 (28) | 18 (39) | 9 (20) |
| Region | Africa (n=32) | 2 (6) | 7 (22) | 14 (44) | 9 (28) |
|  | Asia-Pacific (n=14) | 4 (29) | 6 (43) | 4 (29) | 0 (0) |
| Service level | Primary/secondary (n=22) | 1 (5) | 8 (36) | 9 (41) | 4 (18) |
|  | Tertiary (n=24) | 5 (21) | 5 (21) | 9 (38) | 5 (21) |
| **Environmental and engineering controls** | | | | | |
| **Physical distancing in clinic premises** | | | |  |  |
| Overall (n=46) | | 37 (80) | 8 (17) | 1 (2) | 0 (0) |
| Region | Africa (n=32) | 26 (81) | 6 (19) | 0 (0) | 0 (0) |
|  | Asia-Pacific (n=14) | 11 (79) | 2 (14) | 1 (7) | 0 (0) |
| Service level | Primary/secondary (n=22) | 18 (82) | 4 (18) | 0 (0) | 0 (0) |
|  | Tertiary (n=24) | 19 (79) | 4 (17) | 1 (4) | 0 (0) |
| **Natural ventilation** | |  |  |  |  |
| Overall (n=46) | | 4 (9) | 11 (24) | 26 (57) | 5 (11) |
| Region | Africa (n=32) | 2 (6) | 8 (25) | 19 (59) | 3 (9) |
|  | Asia-Pacific (n=14) | 2 (14) | 3 (21) | 7 (50) | 2 (14) |
| Service level | Primary/secondary (n=22) | 0 (0) | 6 (27) | 15 (68) | 1 (5) |
|  | Tertiary (n=24) | 4 (17) | 5 (21) | 11 (46) | 4 (17) |
| **Precautions for staff members** | | | |  |  |
| **Face shields** | |  |  |  |  |
| Overall (n=46) | | 25 (54) | 6 (13) | 3 (7) | 12 (26) |
| Region | Africa (n=32) | 16 (50) | 3 (9) | 2 (6) | 11 (34) |
|  | Asia-Pacific (n=14) | 9 (64) | 3 (21) | 1 (7) | 1 (7) |
| Service level | Primary/secondary (n=22) | 12 (55) | 2 (9) | 2 (9) | 6 (27) |
|  | Tertiary (n=24) | 13 (54) | 4 (17) | 1 (4) | 6 (25) |
| **Protective clothes** | |  |  |  |  |
| Overall (n=46) | | 20 (43) | 8 (17) | 9 (20) | 9 (20) |
| Region | Africa (n=32) | 13 (41) | 4 (13) | 7 (22) | 8 (25) |
|  | Asia-Pacific (n=14) | 7 (50) | 4 (29) | 2 (14) | 1 (7) |
| Service level | Primary/secondary (n=22) | 12 (55) | 3 (14) | 4 (18) | 3 (14) |
|  | Tertiary (n=24) | 8 (33) | 5 (21) | 5 (21) | 6 (25) |
| **Face masks** | |  |  |  |  |
| Overall (n=46) | | 19 (41) | 20 (43) | 7 (15) | 0 (0) |
| Region | Africa (n=32) | 17 (53) | 12 (38) | 3 (9) | 0 (0) |
|  | Asia-Pacific (n=14) | 2 (14) | 8 (57) | 4 (29) | 0 (0) |
| Service level | Primary/secondary (n=22) | 10 (45) | 8 (36) | 4 (18) | 0 (0) |
|  | Tertiary (n=24) | 9 (38) | 12 (50) | 3 (13) | 0 (0) |
| **Safety glasses** | |  |  |  |  |
| Overall (n=46) | | 19 (41) | 8 (17) | 2 (4) | 17 (37) |
| Region | Africa (n=32) | 12 (38) | 4 (13) | 1 (3) | 15 (47) |
|  | Asia-Pacific (n=14) | 7 (50) | 4 (29) | 1 (7) | 2 (14) |
| Service level | Primary/secondary (n=22) | 11 (50) | 2 (9) | 1 (5) | 8 (36) |
|  | Tertiary (n=24) | 8 (33) | 6 (25) | 1 (4) | 9 (38) |
| **Surgical hoods** | |  |  |  |  |
| Overall (n=46) | | 15 (33) | 6 (13) | 3 (7) | 22 (48) |
| Region | Africa (n=32) | 9 (28) | 3 (9) | 2 (6) | 18 (56) |
|  | Asia-Pacific (n=14) | 6 (43) | 3 (21) | 1 (7) | 4 (29) |
| Service level | Primary/secondary (n=22) | 6 (27) | 1 (5) | 1 (5) | 14 (64) |
|  | Tertiary (n=24) | 9 (38) | 5 (21) | 2 (8) | 8 (33) |
| **Instruction on respiratory etiquette** | | | |  |  |
| Overall (n=46) | | 8 (17) | 16 (35) | 21 (46) | 1 (2) |
| Region | Africa (n=32) | 8 (25) | 11 (34) | 13 (41) | 0 (0) |
|  | Asia-Pacific (n=14) | 0 (0) | 5 (36) | 8 (57) | 0 (0) |
| Service level | Primary/secondary (n=22) | 6 (27) | 8 (36) | 8 (36) | 0 (0) |
|  | Tertiary (n=24) | 2 (8) | 8 (33) | 13 (54) | 1 (4) |
| **Hand sanitation using a disinfectant solution** | | | | |  |
| Overall (n=46) | | 8 (17) | 25 (54) | 13 (28) | 0 (0) |
| Region | Africa (n=32) | 8 (25) | 17 (53) | 7 (22) | 0 (0) |
|  | Asia-Pacific (n=14) | 0 (0) | 8 (57) | 6 (43) | 0 (0) |
| Service level | Primary/secondary (n=22) | 4 (18) | 11 (50) | 7 (32) | 0 (0) |
|  | Tertiary (n=24) | 4 (17) | 14 (58) | 6 (25) | 0 (0) |
| **Protective gloves** | |  |  |  |  |
| Overall (n=46) | | 6 (13) | 14 (31) | 22 (48) | 4 (9) |
| Region | Africa (n=32) | 2 (6) | 7 (23) | 20 (63) | 3 (10) |
|  | Asia-Pacific (n=14) | 4 (29) | 7 (50) | 2 (14) | 1 (7) |
| Service level | Primary/secondary (n=22) | 2 (10) | 6 (29) | 14 (64) | 0 (0) |
|  | Tertiary (n=24) | 4 (17) | 8 (33) | 8 (33) | 4 (17) |
| **Hand hygiene using water and soap** | | | |  |  |
| Overall (n=46) | | 2 (4) | 17 (37) | 27 (59) | 0 (0) |
| Region | Africa (n=32) | 2 (6) | 10 (31) | 20 (63) | 0 (0) |
|  | Asia-Pacific (n=14) | 0 (0) | 7 (50) | 7 (50) | 0 (0) |
| Service level | Primary/secondary (n=22) | 1 (5) | 7 (32) | 14 (64) | 0 (0) |
|  | Tertiary (n=24) | 1 (4) | 10 (42) | 13 (54) | 0 (0) |
| **Precautions for patients** | | |  |  |  |
| **Room cleaning and disinfection after each presumptive or confirmed COVID-19 case** | | | | | |
| Overall (n=46) | | 29 (63) | 7 (15) | 4 (9) | 6 (13) |
| Region | Africa (n=32) | 23 (72) | 2 (6) | 1 (3) | 6 (19) |
|  | Asia-Pacific (n=14) | 6 (43) | 5 (36) | 3 (21) | 0 (0) |
| Service level | Primary/secondary (n=22) | 14 (64) | 1 (5) | 1 (5) | 6 (27) |
|  | Tertiary (n=24) | 15 (63) | 6 (25) | 3 (13) | 0 (0) |
| **Face masks** | |  |  |  |  |
| Overall (n=46) | | 24 (52) | 14 (30) | 8 (17) | 0 (0) |
| Region | Africa (n=32) | 21 (66) | 6 (19) | 5 (16) | 0 (0) |
|  | Asia-Pacific (n=14) | 3 (21) | 8 (57) | 3 (21) | 0 (0) |
| Service level | Primary/secondary (n=22) | 14 (64) | 3 (14) | 5 (23) | 0 (0) |
|  | Tertiary (n=24) | 10 (42) | 11 (46) | 3 (13) | 0 (0) |
| **Hand sanitation using a disinfectant solution** | | | | |  |
| Overall (n=46) | | 22 (48) | 13 (28) | 8 (17) | 3 (7) |
| Region | Africa (n=32) | 19 (59) | 7 (22) | 3 (9) | 3 (9) |
|  | Asia-Pacific (n=14) | 3 (21) | 6 (43) | 5 (36) | 0 (0) |
| Service level | Primary/secondary (n=22) | 12 (55) | 6 (27) | 3 (14) | 1 (5) |
|  | Tertiary (n=24) | 10 (42) | 7 (29) | 5 (21) | 2 (8) |
| **Hand hygiene using water and soap** | | | |  |  |
| Overall (n=46) | | 14 (13) | 14 (28) | 17 (39) | 1 (20) |
| Region | Africa (n=32) | 12 (38) | 9 (28) | 11 (34) | 0 (0) |
|  | Asia-Pacific (n=14) | 2 (14) | 5 (36) | 6 (43) | 1 (7) |
| Service level | Primary/secondary (n=22) | 7 (32) | 5 (23) | 10 (45) | 0 (0) |
|  | Tertiary (n=24) | 7 (29) | 9 (38) | 7 (29) | 1 (4) |
| **Surface cleaning with disinfectant** | | | |  |  |
| Overall (n=46) | | 11 (24) | 21 (47) | 11 (24) | 2 (4) |
| Region | Africa (n=32) | 10 (32) | 11 (35) | 8 (26) | 2 (6) |
|  | Asia-Pacific (n=14) | 1 (7) | 10 (71) | 3 (21) | 0 (0) |
| Service level | Primary/secondary (n=22) | 7 (33) | 9 (43) | 5 (24) | 0 (0) |
|  | Tertiary (n=24) | 4 (17) | 12 (50) | 6 (25) | 2 (8) |
| **Instruction on respiratory etiquette** | | | |  |  |
| Overall (n=46) | | 9 (20) | 16 (35) | 20 (43) | 1 (2) |
| Region | Africa (n=32) | 8 (25) | 10 (31) | 14 (44) | 0 (0) |
|  | Asia-Pacific (n=14) | 1 (7) | 6 (43) | 6 (43) | 1 (7) |
| Service level | Primary/secondary (n=22) | 5 (23) | 8 (36) | 9 (41) | 0 (0) |
|  | Tertiary (n=24) | 4 (17) | 8 (33) | 11 (46) | 1 (4) |
| **Surface cleaning with water and/or soap** | | | | |  |
| Overall (n=46) | | 8 (17) | 18 (39) | 20 (43) | 0 (0) |
| Region | Africa (n=32) | 8 (25) | 11 (34) | 13 (41) | 0 (0) |
|  | Asia-Pacific (n=14) | 0 (0) | 7 (50) | 7 (50) | 0 (0) |
| Service level | Primary/secondary (n=22) | 3 (14) | 8 (36) | 11 (50) | 0 (0) |
|  | Tertiary (n=24) | 5 (21) | 10 (42) | 9 (38) | 0 (0) |

**Supplementary Figure 1:** Detailed overview of impacts on any health services and resources during the COVID-19 pandemic at the 46 participating ART clinics (situation as of July 2021). The pattern of each clinic is shown.


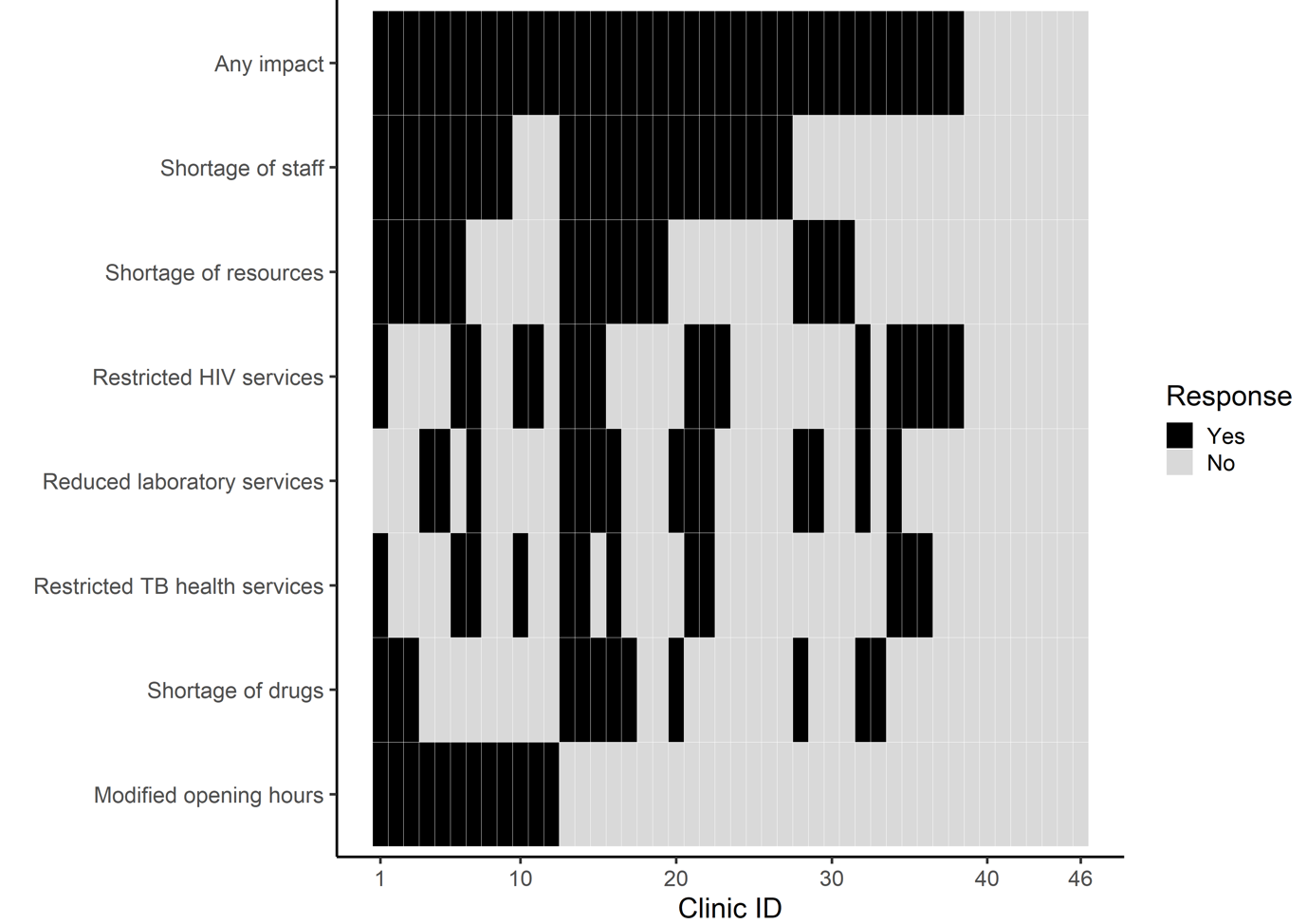


TB, tuberculosis

**Supplementary Figure 2:** Detailed overview of control measures for staff and patients.


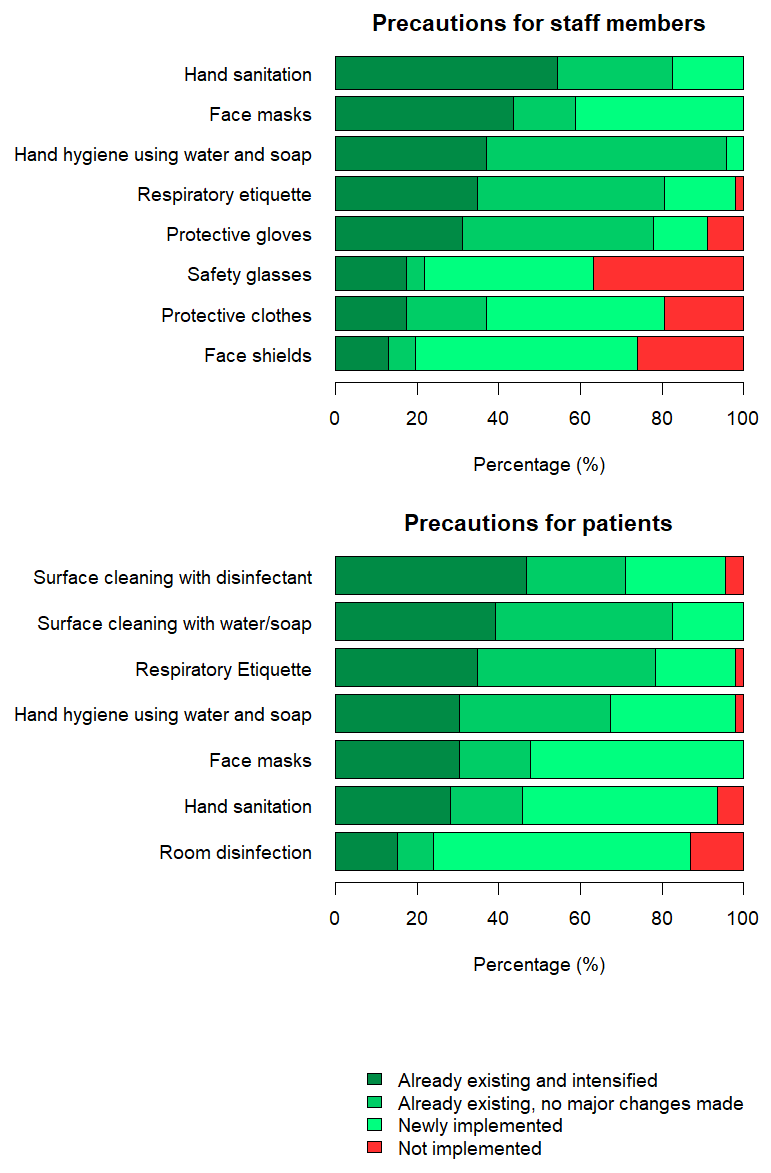

Supplement: Supplementary file 1 — Figure S1: Detailed overview of impacts on any health services and resources during the COVID‐19 pandemic at the 46 participating ART clinics (situation as of July 2021). Figure S2: Detailed overview of control measures for staff and patients. Table S1: Site description of the 46 participating antiretroviral therapy (ART) clinics offering tuberculosis services. Table S2: Diagnostic practices of SARS‐CoV‐2 at the ART clinics (situation as of July 2021). Table S3: Trends in tuberculosis (TB) diagnoses over time (based on data from 37/46 participating clinics). Table S4: Overview of infection control measures at the ART clinics. [file JIA2-25-e26018-s001.docx]
